# Meteorological Register

**Published:** 1814-01

**Authors:** C. Blunt

**Affiliations:** Philosophical Instrument Maker, No. 38, Tavistock-Street, Covent-Garden


### METEOROLOGICAL REGISTER.

*From November 25, to December 25,* 1813.

**Kept by C. BLUNT,** Philosophical Instrument Maker, No. 38, Tavistock-Stieet, Covent-Garden.

| Moon. | Day. | Wind. | Barometrical Pressure. | | Temperature. | | |
|---|---|---|---|---|---|---|---|
| | | | Max. | Min. | Max. | Min. | |
| | 26 | SE | 30·23 | 30·10 | 47 | 42 | Fair |
| | 27 | W | 29 32 | 29·26 | 49 | 45 | Fair |
| | 28 | SW | 30·24 | 29 48 | 50 | 44 | Cloudy |
| | 29 | W | 29·75 | 29·50 | 51 | 42 | Cloudy |
| | 30 | W | 29·27 | 29 03 | 49 | 31 | Fair |
| ◐ | 1 | E | 29·47 | 29 42 | 36 | 34 | Fair |
| | 2 | SE | 29·31 | 29 07 | 37 | 36 | Rain |
| | 3 | SE | 29·24 | 29·05 | 42 | 39 | Rain |
| | 4 | EbN | 29 47 | 29·33 | 44 | 39 | Foggy |
| | 5 | E | 29 56 | 29·50 | 41 | 39 | Foggy |
| | 6 | NE | 29 79 | 29·74 | 41 | 39 | Fair |
| ○ | 7 | N | 29·79 | 29·78 | 41 | 39 | Fair |
| | 8 | N | 29 81 | 29·77 | 42 | 41 | Fair |
| | 9 | N | 29 94 | 29·88 | 41 | 40 | Foggy |
| | 10 | NE | 30·10 | 30·08 | 40 | 38 | Fair |
| | 11 | E | 30·19 | 30·06 | 37 | 35 | Fair |
| | 12 | E | 30 79 | 29·87 | 35 | 32 | Fair |
| | 13 | E | 29 83 | 29 74 | 32 | 30 | Cloudy |
| ◑ | 14 | SE | 29 92 | 29 90 | 30 | 26 | Fair |
| | 15 | SW | 29 87 | 29·70 | 32 | 26 | Fair |
| | 16 | W | 29·50 | 29 40 | 47 | 32 | Rain |
| | 17 | SW | 29·44 | 29·27 | 50 | 47 | Rain |
| | 18 | SW | 29 16 | 29·14 | 55 | 49 | Rain |
| | 19 | SW | 29 20 | 29·15 | 51 | 44 | Cloudy |
| | 20 | W | 29 42 | 29·29 | 43 | 35 | Fair |
| | 21 | W | 29 61 | 29·46 | 40 | 32 | Rain |
| ● | 22 | NW | 29·55 | 29·41 | 46 | 42 | Fair |
| | 23 | SW | 29·92 | 29·70 | 44 | 37 | Fair |
| | 24 | SW | 30 04 | 29·92 | 50 | 44 | Cloudy |
| | 25 | W | 30·07 | 30 03 | 50 | 46 | Cloudy |

Mean Height of the Barometer, 29·655.—Mean Temperature, 40·64.

